# High Prevalence of Cefiderocol Resistance Among New Delhi Metallo-β-Lactamase Producing *Klebsiella pneumoniae* High-Risk Clones in Hungary

**DOI:** 10.3390/antibiotics14050475

**Published:** 2025-05-08

**Authors:** Lilla Buzgó, Zsanett Kiss, Dániel Göbhardter, Virág Lesinszki, Erika Ungvári, Zoltán Rádai, Levente Laczkó, Ivelina Damjanova, Gábor Kardos, Ákos Tóth

**Affiliations:** 1 Department of Bacteriology, Parasitology and Mycology, National Center for Public Health and Pharmacy, 1097 Budapest, Hungary; kisszsani95@gmail.com (Z.K.); gobhardter.daniel@nngyk.gov.hu (D.G.); lesinszki.virag@nngyk.gov.hu (V.L.); ungvari.erika@nngyk.gov.hu (E.U.); damjanova.ivelina@nngyk.gov.hu (I.D.); kardos.gabor@nngyk.gov.hu (G.K.); toth.akos@nngyk.gov.hu (Á.T.); 2 One Health Institute, Faculty of Health Sciences, University of Debrecen, 4032 Debrecen, Hungary; radai.zoltan@med.unideb.hu (Z.R.); laczko.levente@etk.unideb.hu (L.L.); 3 Department of Dermatology, Medical Faculty and University Hospital Duesseldorf, Heinrich-Heine-University Duesseldorf, 40225 Duesseldorf, Germany; 4 HUN-REN-UD Conservation Biology Research Group, University of Debrecen, 4032 Debrecen, Hungary; 5 Institute of Metagenomics, University of Debrecen, 4032 Debrecen, Hungary

**Keywords:** high-risk clones, carbapenemase, *K. pneumoniae*, resistance, cefiderocol

## Abstract

Background/Objectives: The global spread of carbapenemase-producing *K. pneumoniae* (CPKP) strains represent a severe public health threat due to very limited choice of antibacterial therapy. Cefiderocol, a novel siderophore-cephalosporin, may represent a new therapeutic option but resistance is increasingly being described. Our aim was to investigate in vitro cefiderocol susceptibility among CPKP strains in Hungary and assess correlations between resistance, carbapenemase types, and clonal lineages. Methods: The study was performed on 420 CPKP strains from 34 Hungarian healthcare institutes (HCIs) submitted to the National Reference Laboratory of Antimicrobial Resistance (March 2021 to April 2023). The disk diffusion method (Liofilchem, Via Scozia, Italy) was used for in vitro cefiderocol susceptibility testing (according to EUCAST guidelines). For molecular epidemiologic investigation, we used whole genome sequencing (Illumina MiSeq, 150 bp paired-end) and pulsed-field gel electrophoresis (PFGE). Carbapenemase gene type was determined by multiplex PCR. Statistical analysis was performed in R (v.4.2.0). Results: Dominant high-risk clones (ST147, ST395, ST258) exhibited regional distribution, with ST147/NDM-1 strains showing the highest cefiderocol resistance (75%). Overall resistance was 65%. Carbapenemase gene types occurred as follows: 35 *bla*_VIM_, 53 *bla*_KPC_, 57 *bla*_OXA-48-like_, 153 *bla*_NDM_, and 122 *bla*_OXA-48-like_+*bla*_NDM_. Cefiderocol resistance rates by carbapenemase type were 20%, 44%, 70%, and 75% in the case of *bla*_VIM_, *bla*_OXA-48-like_, *bla*_KPC_, *bla*_NDM,_ and *bla*_OXA-48-like_+*bla*_NDM_. Conclusions: The results show a high prevalence of cefiderocol resistance in CPKP in Hungary, with different rates of resistance in different carbapenemase gene-carrying high-risk clones, highlighting the growing challenge in treating these infections.

## 1. Introduction

In recent years, the spread of carbapenemase-producing *Enterobacterales* (CPEs) has become one of the main global public health threats. CPEs have been detected in many countries across Europe, with variable abundance and clonal distribution according to geographical location [1]. Among CPEs, *Klebsiella pneumoniae* stands out for rapid spread and high resilience [2].

The high levels of resistance to third-generation cephalosporins used in the treatment of *K. pneumoniae* infections has led to the increased use of carbapenems as the “last resort” agent. This may have enhanced the selection of potential successful bacterial clones and the carbapenem-resistant *K. pneumoniae* (CRKP) clones becoming prevalent across all regions of the world [3,4]. The severity of antimicrobial resistance (AMR) is reflected in the global spread of high-risk clones and/or the movement of mobile genetic elements linked to AMR between different clones, as AMR spreads through these clones [4,5].

The key mechanism of carbapenem resistance in *Klebsiella pneumoniae* is the production of carbapenemase enzymes (the most prevalent carbapenemase types are Klebsiella pneumoniae carbapenemase (KPC), New Delhi metallo-β-lactamase (NDM), Verona integron-encoded metallo-β-lactamase (VIM), and Oxacillinase (OXA)-48-like), so high-risk clones such as KPC-type carbapenemase-producing CC258 (clonal complex 258) clone and OXA-48-like-type carbapenemase-producing ST395 or ST15 clones have facilitated the worldwide spread of carbapenemases [1,5,6].

In Europe, multidrug-resistant *K. pneumoniae* and/or CRKP strains are reported to be responsible for >90,000 infections, >7000 deaths per year, and 25% of all disability-adjusted life-years (DALYs) lost [3,5,7]. In 2022, prevalence of carbapenem resistance among *K. pneumoniae* isolated from blood cultures in some European countries has risen to alarming levels, for example 72% in Greece, 47.3% in Bulgaria, and 47.8% in Romania [8].

A major difficulty in the treatment of infections caused by CRKP strains is that these are usually multidrug-resistant and/or extensively drug-resistant, possibly pandrug-resistant [9]. The WHO recently identified carbapenem-resistant *Enterobacterales*, including CRKP, as one of the three bacterial complexes for which there is a critical need to develop new antibiotics [10]. With these the therapeutic limitations, it is very important to extensively test new antimicrobial agents that are available, such as cefiderocol. In Hungary, the isolation rate of CRKP in bloodstream infections has increased by 744% by 2023 compared to 2019. This concerning situation shows how important it is to assess the susceptibility of the Hungarian CRKP population to cefiderocol [11].

Since becoming commercially available, the new β-lactam siderophore-cephalosporin, called cefiderocol, has been reported to be effective against Gram-negative bacteria producing ß-lactamases of Ambler classes A, B, C, and D [12,13,14,15,16,17]. It accumulates in the periplasmic space and inhibits cell wall synthesis due to its novel penetration mechanism (via active iron transport through siderophore receptors), which is different from previously known β-lactam antibiotics and provides faster uptake [13,15]. Recently, however, concerns have been raised about cefiderocol use. It should only be used for the treatment of complicated urinary tract infections, hospital-acquired pneumonia, and ventilator-associated pneumonia. It was also found that treatment with cefiderocol was associated with an increase in all-cause mortality compared to the best available therapy [6,18,19].

The aim of our study was to investigate the in vitro cefiderocol susceptibility of CPKP strains prevalent in Hungary, and the correlation between the occurrence of cefiderocol resistance and specific carbapenemase types and CPKP clones.

## 2. Results

### 2.1. Cefiderocol Susceptibility of CPKP Strains by Carbapenemase Types

#### 2.1.1. Prevalence of Carbapenemase Genes (by Polymerase Chain Reaction (PCR)) and Cefiderocol Resistance Patterns

**Figure 1:**
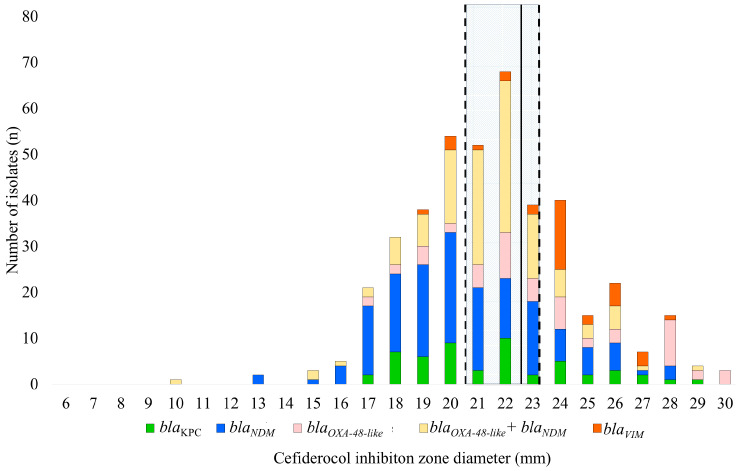
Distribution of the cefiderocol inhibition zone diameters (mm) by susceptibility category and carbapenemase type. The part between dashed lines indicates the ATU category. The solid line marks the clinical breakpoint (mm) between the susceptibility categories. A cefiderocol inhibition zone diameter ≥23 mm corresponds to the S (Susceptible, standard dosing regimen) category, while an inhibition zone diameter <23 mm corresponds to the R (resistant) category.

The sole presence of 35 *bla*_VIM_, 53 *bla*_KPC_, 57 *bla*_OXA-48-like_, and 153 *bla*_NDM_ and co-presence of 122 *bla*_OXA-48-like_ and *bla*_NDM_ (hereafter referred to as double carbapenemases and marked with a + sign, e.g., *bla*_OXA-48-like_+*bla*_NDM_) carbapenemase genes were detected by multiplex PCR among the studied strains (n = 420). The overall cefiderocol resistance rate was 65%; resistance rates were 20%, 44%, 70%, and 75% for *bla*_VIM_-carrying, *bla*_OXA-48-like_-carrying, *bla*_KPC_-carrying isolates, *bla*_NDM_-carrying, and double carbapenemase carriers, respectively. The overall proportion of isolates in the ATU (Area of Technical Uncertainty—a value for which interpretation difficulties arise when the test is performed correctly and a fully reproducible interpretation is not possible) category based on cefiderocol inhibition zone diameters was 38% (Figure 1); 14%, 28%, 31%, 35%, and 59% of *bla*_VIM_-carrying, *bla*_KPC_-carrying, *bla*_NDM_-carrying *bla*_OXA-48-like_-carrying, and double carbapenemase-carrying isolates, respectively, fell into the ATU category according to the zone diameters (Table 1).

**Figure 2:**
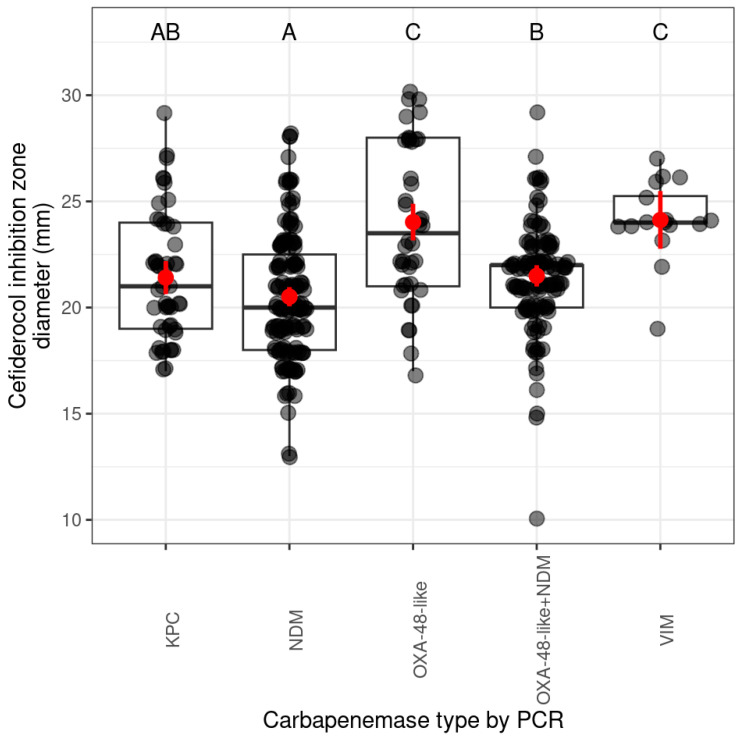
Distribution of cefiderocol inhibition zones (mm) by carbapenemase types. The scale on the left side of the figure shows the diameter (mm) of the inhibition zone of cefiderocol. The lower part of the figure shows the types of carbapenemase genes (by PCR) produced by the tested strains. The letters in the upper part of the figure indicate the statistical significance of the differences between the cefiderocol inhibition zone of the carbapenemase types (different letters indicate significantly different groups). The grey dots represent the individual isolates. If several isolates have the same diameter, the dots overlap and appear darker/black. The horizontal line in the box classically indicates the mean of the presented values, in our case the mean diameter of the cefiderocol inhibition zones (mm); the red dots show the estimated marginal mean of the cefiderocol inhibition zone diameter (mm) per group with 95% confidence interval (red bars).

Inhibition zone diameters of the *bla*_VIM_-carrying and *bla*_OXA-48-like_-carrying isolates did not differ significantly. These isolates had the largest mean of cefiderocol inhibition zone diameter of cefiderocol, indicating low resistance to this antibiotic. The strains carrying *bla*_OXA-48-like_+*bla*_NDM_ had significantly lower means of inhibition zone diameters (mm) than *bla*_VIM_-carrying or *bla*_OXA-48-like_-carrying strains, indicating higher resistance to cefiderocol. Significant difference was also observed between the *bla*_OXA-48-like_+*bla*_NDM_-carrying and *bla*_NDM_-carrying strains. The strains harbouring *bla*_KPC_ did not differ significantly from *bla*_NDM_-carrying and *bla*_OXA-48-like_+*bla*_NDM_-carrying strains. Notably, the lowest mean of inhibition zone diameter was observed for strains carrying *bla*_NDM_ and *bla*_KPC_ (Figure 2, more details in Supplementary Table S2), indicating the highest resistance to cefiderocol.

#### 2.1.2. Prevalence of Cefiderocol Resistance Among PFGE Pulsotypes and Their Related Carbapenemase Genes

**Figure 3:**
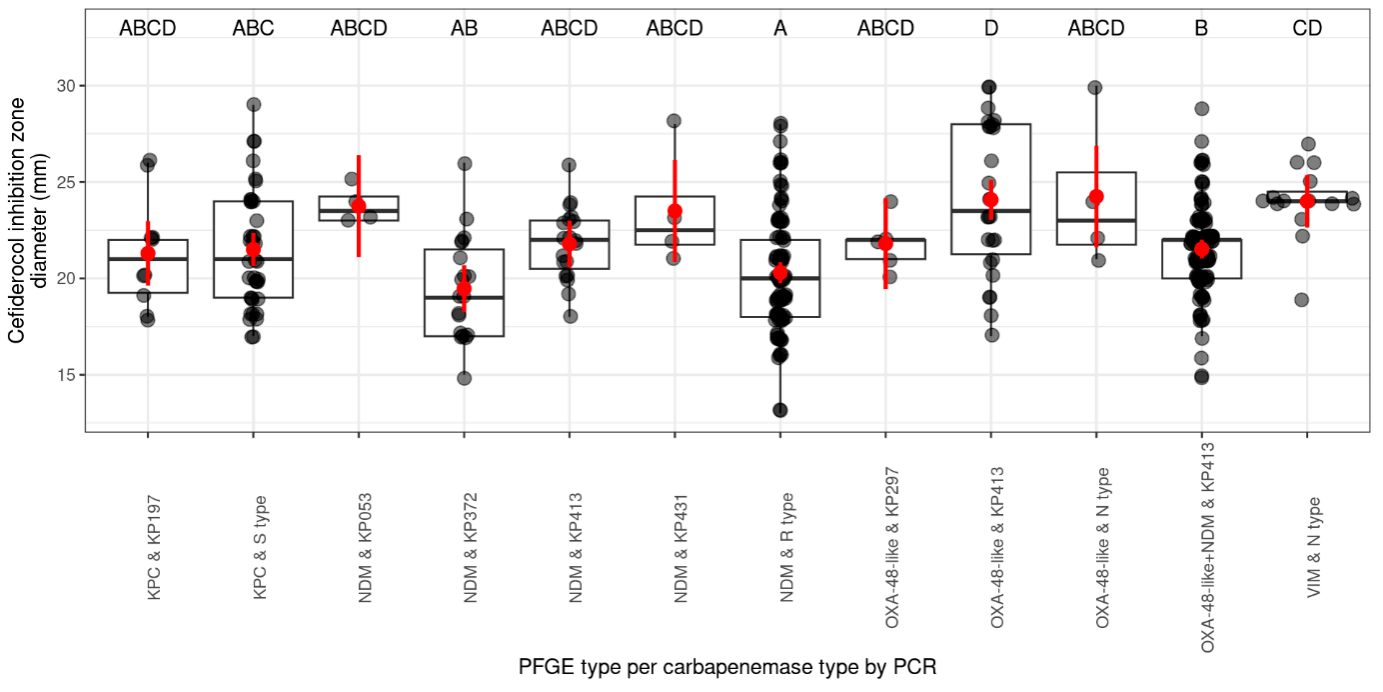
Distribution of cefiderocol inhibition zones (mm) by PFGE type per carbapenemase. The scale on the left side of the figure shows the diameter (mm) of the inhibition zone of cefiderocol. The lower part of the figure shows the combination of the indicated pulsotype and the carbapenemase type (by PCR). The letters in the upper part of the figure indicate the statistical significance of the differences between the cefiderocol inhibition zone of the PFGE types and the carbapenemase type combination (different letters indicate significantly different groups). The grey dots represent the individual isolates. If several isolates have the same diameter, the dots overlap and appear darker/black. The horizontal line in the box classically indicates the mean of the presented values, in our case the mean diameter of the cefiderocol inhibition zones (mm); the red dots show the estimated marginal mean of the cefiderocol inhibition zone diameter (mm) per group with 95% confidence interval (red bars).

In the PFGE analysis, 39 different pulsotypes were detected. The cefiderocol inhibition zone diameters of nine pulsotypes per carbapenemase type (by PCR) were compared in total. The majority of cefiderocol inhibition zone diameters (mm) of the pulsotype–carbapenemase combinations included in the comparison did not show significant differences from each other. The pulsotype–carbapenemase combinations that were significantly different from each other were as follows: *bla*_OXA-48-like_ and KP413, *bla*_OXA-48-like_+*bla*_NDM_ and KP413, *bla*_NDM_ and KP372, *bla*_VIM_ and N pulsotype, and *bla*_NDM_ and R pulsotype. The *bla*_NDM_-producing KP372 and *bla*_NDM_-producing R pulsotype isolates had the lowest mean of cefiderocol inhibition zone diameter (mm). The *bla*_OXA-48-like_-producing and *bla*_VIM_-producing N pulsotype and also the *bla*_OXA-48-like_-producing KP413 pulsotype strains had the largest means of cefiderocol inhibition zone (Figure 3, more details in Supplementary Table S3).

#### 2.1.3. Occurrence of Cefiderocol Resistance Between ST (Sequence Type) Based on PFGE Pulsotypes

We assigned STs to the PFGE pulsotypes of the strains (n = 275) without MLST (Multi-Locus Sequence Typing) from our database. The sequence type could not be inferred for 40 isolates. We classified the strains into seven PFGE-based STs (more details in Supplementary Table S1).

**Figure 4:**
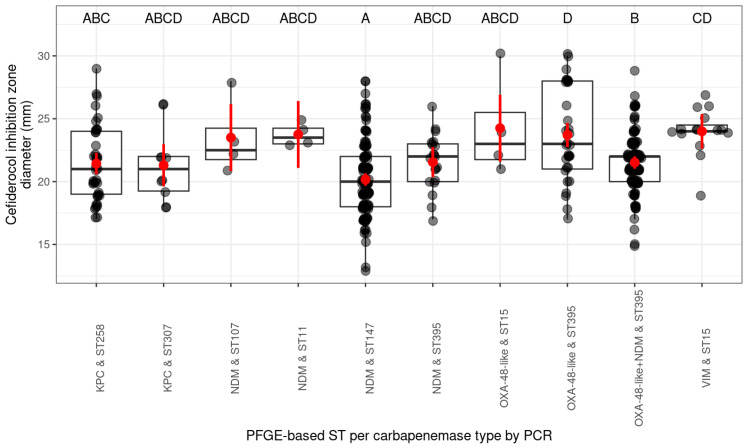
Distribution of cefiderocol inhibition zones (mm) by PFGE-based ST per carbapenemase (by PCR) and significance level plot between types (95% confidence interval). The scale on the left side of the figure shows the diameter (mm) of the inhibition zone of cefiderocol. The lower part of the figure shows the combination of the indicated PFGE-based ST and the carbapenemase type (by PCR). The letters in the upper part of the figure indicate the statistical significance of the differences between the cefiderocol inhibition zone of the PFGE-based types and the carbapenemase type combination (different letters indicate significantly different groups). The grey dots represent the individual isolates. If several isolates have the same diameter, the dots overlap and appear darker/black. The horizontal line in the box classically indicates the mean of the presented values, in our case the mean diameter of the cefiderocol inhibition zones (mm); the red dots show the estimated marginal mean of the cefiderocol inhibition zone diameter (mm) per group with 95% confidence interval (red bars).

Ten PFGE-based ST type–carbapenemase combinations were included in the statistical analysis. The cefiderocol inhibition zone diameters (mm) of the PFGE-based ST type and carbapenemase (PCR-based) combinations (*bla*_KPC_ and ST307, *bla*_NDM_ and ST107, *bla*_NDM_ and ST11, *bla*_NDM_ and ST395, *bla*_OXA-48-like_ and ST15) did not differ significantly from the other combinations in this comparison. The combination of *bla*_NDM_ and ST147, *bla*_OXA-48-like_ and ST395, *bla*_OXA-48-like_+ *bla*_NDM_ and ST395, *bla*_VIM_ and ST15, and *bla*_KPC_ and ST258 differed significantly from other PFGE-based ST type–carbapenemase combinations. Significant differences were observed for the combinations *bla*_NDM_ and ST147, *bla*_OXA-48-like_ and ST395, *bla*_OXA-48-like_+ *bla*_NDM_ and ST395, *bla*_VIM_ and ST15, and *bla*_KPC_ and ST258. The lowest mean cefiderocol inhibition zone diameter (mm) was observed for *bla*_NDM_-producing ST147. The *bla*_OXA-48-like_-producing ST15 and *bla*_VIM_-producing ST15 strains had the largest mean cefiderocol inhibition zone (Figure 4, more details in Supplementary Table S4).

#### 2.1.4. Overview of the Frequency of Cefiderocol Resistance Based on the cgMLST (Core-Genome MLST) Results

**Figure 5:**
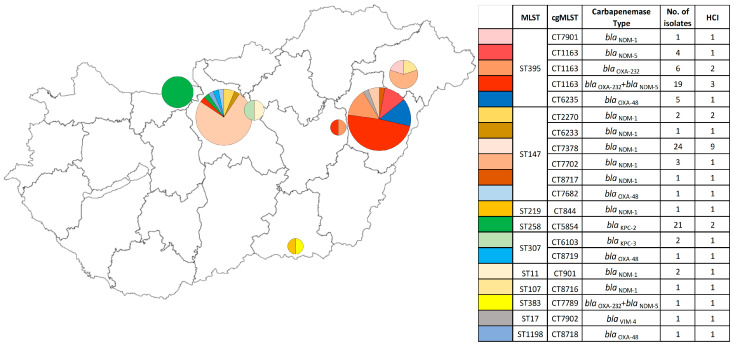
Geographical distribution of studied CPKP clones in Hungary based on cgMLST. The different colours in the figure represent the ST and CT types, which are indicated in the columns “MLST” and “cgMLST”. The size of the pie charts corresponds to the number of isolates. The column “Carbapenemase Type” shows the carbapenemase types determined by sequence analysis. The “HCI” column contains the number of submitting healthcare institutions.

Among the 98 CPKP isolates from 34 HCIs genotyped with cgMLST, 35 belonged to sequence type ST395 (three complex types (CTs)), 32 to sequence type ST147 (five CTs), and 21 to CC258 (one CT). From the remaining 10 isolates, three belonged to sequence type ST307 (two CTs), two isolates to sequence type ST11 (one CT), and one isolate each to ST107 (one CT), ST219 (one CT), ST17 (one CT), ST383 (one CT), and ST1198 (one CT). The CTs of the isolates and the carbapenemase genes they carry are shown in Figure 5.

Based on WGS (Whole Genome Sequencing) analysis, *bla*_NDM-1_ (n = 36) and *bla*_NDM-5_ (n = 24) were the prevalent *bla*_NDM_ types. Of these, 19 cases of NDM-5 occurred in association with OXA-232 and one case with OXA-48 carbapenemases. The population shows a segregation of these groups, with 89% of strains carrying *bla*_NDM-1_ belonging to the ST147 sequence type and 96% of strains carrying *bla*_NDM-5_ belonging to the ST395 sequence type. Among the KPC enzymes, *bla*_KPC-2_ (n = 21) belonging to ST258 was found to be predominant over *bla*_KPC-3_ (n = 2) belonging to ST307. The *bla*_OXA-232_ and *bla*_OXA-232_+*bla*_NDM-5_ (n = 25) belonging to ST395 occurred in higher numbers than *bla*_OXA-48_ and *bla*_OXA-48_+*bla*_NDM-5_ (n = 9) belonging to ST395, ST307, ST147, ST1198, and ST383.

Comparing the sequence type and carbapenemase (WGS-based) combinations by cefiderocol inhibition diameter (mm), there was a significant difference between the *bla*_NDM_-carrying isolates belonging to ST147 and the *bla*_OXA-like_-carrying ST395 isolates. The *bla*_NDM_-carrying ones belonging to ST395 were not significantly different from the latter. We did not find significant differences between the *bla*_OXA-like_-carrying ST395 and the *bla*_KPC_-carrying ST258, *bla*_OXA-like_+ *bla*_NDM_-carrying ST395 strains. Markedly, those belonging to the *bla*_NDM_-carrying strain ST147 and to the *bla*_NDM_-carrying strain ST395 had the smallest mean of cefiderocol inhibition zone.

When analysing the cefiderocol inhibition zone diameter (mm) and ST–CT types, the strains belonging to ST395 CT1163 and ST258 CT5854 were not found to be significantly different. Significantly different from these were those belonging to ST147 CT7378 strains, which had the lowest mean cefiderocol inhibition diameter (mm). Strains belonging to ST395 CT6235 did not differ significantly from other groups.

**Figure 6:**
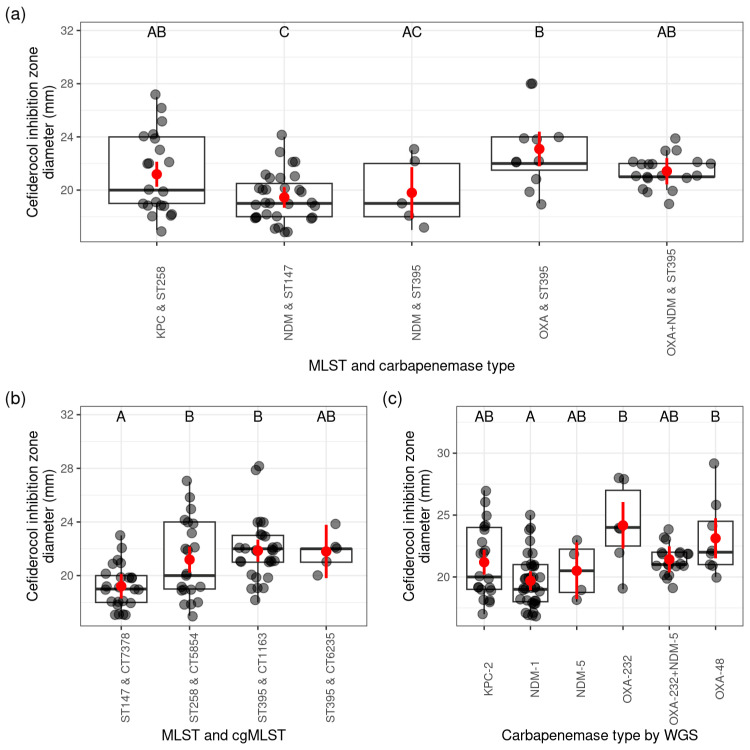
Result of cefiderocol inhibition zone diameters (mm) and results of MLST and cgMLST analysis. The scales on the left sides of the figures show the diameter (mm) of the inhibition zones of cefiderocol. The lower part of figure (**a**) shows the combination of the indicated ST and the carbapenemase gene type (by sequence analysis). The lower part of figure (**b**) shows the combination of ST and CT. The lower part of figure (**c**) shows the carbapenemase genes (by WGS) produced by the studied strains. The letters in the upper part of the figures show the statistical significance of the differences between the cefiderocol inhibition zone of the different groups and carbapenemase type combinations, STs and CTs, and carbapenemase type (different letters indicate significantly different groups. The grey dots represent the individual isolates. If several isolates have the same diameter, the dots overlap and appear darker/black. The horizontal line in the box classically indicates the mean of the presented values, in our case the mean diameter of the cefiderocol inhibition zones (mm); the red dots show the estimated marginal mean of the cefiderocol inhibition zone diameter (mm) per group with 95% confidence interval (red bars).

Furthermore, the cefiderocol inhibition zone diameters (mm) of *bla*_OXA-232_ or *bla*_OXA-48_ enzyme-producing strains did not differ significantly and had the largest cefiderocol inhibition zone diameter (mm). The cefiderocol inhibition zone diameters (mm) of *bla*_NDM-1_-producing isolates were significantly different from these and the lowest. The *bla*_KPC-2_-, *bla*_NDM-5_-, and *bla*_OXA-232_+*bla*_NDM-5_-producing isolates were not significantly different from the other groups (Figure 6, more details in Supplementary Tables S5–S7).

Furthermore, the cefiderocol inhibition zone diameters (mm) of *bla*_OXA-232_ or *bla*_OXA-48_ enzyme-producing strains did not differ significantly and had the largest cefiderocol inhibition zone diameter (mm). The cefiderocol inhibition zone diameters (mm) of *bla*_NDM-1_-producing isolates were significantly different from these and the lowest. The *bla*_KPC-2_-, *bla*_NDM-5_-, and *bla*_OXA-232_+*bla*_NDM-5_-producing isolates were not significantly different from the other groups (Figure 6, more details in Supplementary Tables S5–S7).

## 3. Discussion

Our study was the first comprehensive study of the clonal distribution and carbapenemase types of CPKP strains prevalent in Hungary. The genome-based typing revealed the same population structure as routine PFGE typing, highlighting about a quarter of the population, but yielded more information and better resolution. Both the PFGE- and more specifically the WGS-based typing showed that the CPKP population structure during the study period was mainly characterised by the prevalence of high-risk clones. The predominant sequence type during the study period was ST395 in the eastern part of the country, ST147 in the north-eastern region and the capital city, and ST258 in the north-western region. Unfortunately, limited data were available for the western, southern, and northern parts of the country (more details see Supplementary Figure S2). However, the results of genotyping studies also suggest that the recently identified CPKP high-risk clones have caused several outbreaks in Hungary in recent years.

The spread of the ST395 clone is a major concern. In 2014, an outbreak in Western Hungary was caused by a *bla*_OXA-48_-harbouring ST395 strain. The index case of that outbreak was a Ukrainian patient. In 2022, the *bla*_OXA-48-like_+*bla*_NDM_-harbouring ST395 appeared in Hungary [22]. The emergence of ST395 *bla*_OXA-48-like_+*bla*_NDM_ strains coincides with reports of similar clones in Ukraine suggesting potential cross-border transmission [23,24]. The clone ST147 as an extended spectrum β-lactamase (ESBL)-producer was already present in Hungary in the early 2000s (more than 20% of all ESBL-producing *K. pneumoniae* strains submitted to the National Center for Epidemiology belonged to strain ST147), and *bla*_KPC-2_-harbouring ST258 was first described in 2008 [4,25,26,27].

We observed the dominance of double carbapenemase-carrying clones instead of the previously almost dominant *bla*_VIM-4_-carrying ST15 clone in Hungary. The homogeneous population structure of CPKP has undergone a major shift due to the spread and dominance of newly emerging high-risk clones [28]. In our study, the dominance of *bla*_NDM_ and *bla*_OXA-48-like_+*bla*_NDM_-carrying clones was observed in Hungary. The prevalence of the NDM enzyme has increased in recent years and regional or interregional spread has been reported throughout the world. Also, an increasing number of countries are reporting OXA-48-like+NDM-producing extensively drug-resistant CPKP strains [6,29,30,31,32,33]. This is of particular concern because among the newly approved β-lactam/β-lactamase inhibitor combinations ceftazidime–avibactam, meropenem–vaborbactam, and imipenem–cilastatin–relebactam are inactive against metallo-β-lactamases such as NDM [6].

Furthermore, results of our cefiderocol disk diffusion susceptibility testing have shown that cefiderocol resistance is present in the Hungarian CPKP population. We found that the *bla*_OXA-48-like_-carrying and *bla*_VIM_-carrying strains had the lowest cefiderocol resistance rate and the largest cefiderocol inhibition zone diameter (mm). The *bla*_KPC_-carrying strains had smaller mean cefiderocol inhibition zone diameters than *bla*_OXA-48-like_+*bla*_NDM_-carrying strains. Nevertheless, the *bla*_OXA-48-like_+*bla*_NDM_-carrying strains had a higher cefiderocol resistance rate than the *bla*_KPC_-carrying strains. Although the resistance rates of *bla*_OXA-48-like_+*bla*_NDM_-carrying strains and only *bla*_NDM_-carrying strains were the same, clones carrying only *bla*_NDM_ (especially *bla*_NDM-1_) had the lowest cefiderocol inhibition zone diameters in all cases.

Cefiderocol resistance is increasingly being reported from several countries (especially in NDM-producing bacterial strains). Sandfort et al. (2022) [29] found 80 NDM-producing CPKPs among those reported to the German National Reference Center by October 2022. All representative *K. pneumoniae* isolates tested (n = 9, ST147, ST395, ST307, ST23) were resistant to cefiderocol. When analysing the cefiderocol susceptibility of nine *Enterobacterales*, 28 *Acinetobacter baumannii*, and one *Pseudomonas aeruginosa* strains, Kohira et al. (2020) [34] found that NDM producers had the highest Minimum Inhibitory Concentration (MIC) values. In the carbapenemase-producing *Enterobacter cloacae* isolates analysed by Nurjadi et al. (2022) [35], cefiderocol resistance was observed in the NDM producers. In the SIDERO-CR study 2014–2016, in which 23 European countries participated, among the CPEs strains, 16% of *bla*_KPC_-carrying (n = 238), 12% of *bla*_OXA-48-like_-carrying (n = 85), 20% of *bla*_VIM_-carrying (n = 62), and 49% of *bla*_NDM_-carrying (n = 37) were resistant to cefiderocol [36]. Among CPEs strains, Mushtaq et al. (2020) [37] found that 59% of *bla*_NDM_-carrying strains (n = 92), 20% of *bla*_VIM_-carrying strains (n = 77), 10% of *bla*_KPC_-carrying strains (n = 56), and 7% of *bla*_OXA-48-like_-carrying strains (n = 56) were resistant to cefiderocol. The role of _NDM_-type metallo-β-lactamases in the development of cefiderocol resistance and the higher proportion of resistance compared to other carbapenemases is also supported by our own results in addition to these reports [9,35,38].

However, high rates of cefiderocol resistance are also frequently reported in *bla*_KPC_-producing *Enterobacterales*, often in association with ceftazidime/avibactam co-resistance (especially in *K. pneumoniae*) [38,39,40]. In one study, ceftazidime/avibactam-resistant *K. pneumoniae* strains (n = 40) had 83% cefiderocol resistance, while cefiderocol resistance in ceftazidime/avibactam-susceptible strains (n = 60) was 7%. Cefiderocol resistance has also been described among *Enterobacterales* carrying *bla*_OXA-427_. In vivo resistance to cefiderocol is also gaining precedence [9,38,39,41].

The interpretation and evaluation of cefiderocol resistance data is also complicated by the fact that the European Committee on Antimicrobial Susceptibility Testing (EUCAST) issued a warning in 2022 for freeze-dried panels used for cefiderocol broth microdilution MIC determination, which is still in place [21]. However, many of the studies conducted prior to the warning examined the susceptibility of cefiderocol using the broth microdilution method. For all these reasons, we used the disk diffusion method for our cefiderocol susceptibility testing. In addition, the EUCAST breakpoint values have changed considerably in January 2024, which may significantly affect comparability with previous years [42].The resistance rates determined in our study were also substantially influenced by the change in the breakpoint value compared to the breakpoint values applicable in the year the study was conducted.

Our results indicate that cefiderocol resistance is related to the carbapenemase type and not to the clones, which is confirmed by the fact that several clones carry the same carbapenemase gene. In addition, we did not investigate the mechanisms underlying cefiderocol resistance and other mechanisms related to NDM production [34], but applied statistical methods to test whether molecular epidemiological data could be associated with the results of cefiderocol susceptibility testing. We also found a correlation between PFGE genotyping and genome-based typing in the statistical analysis of the cefiderocol inhibition zone. Furthermore, our study is a retrospective observational analysis limited by the fact that it only includes isolates received at the National Reference Laboratory for Antimicrobial Resistance and not data from the entire country. In addition, we have not investigated the genetic background of cefiderocol resistance. However, we plan to perform long-read sequencing (Nanopore-MinION platform) on some isolates and investigate these mechanisms.

## 4. Materials and Methods

### 4.1. Bacterial Isolates

From March 2021 to April 2023, 420 CPKP non-duplicate isolates obtained from 32 Hungarian healthcare institutes (HCIs) and two general medical practices were mandatorily submitted (legally defined) to the National Reference Laboratory for Antimicrobial Resistance at the National Center for Public Health and Pharmacy (more details see Supplementary Figure S1). A total of 20% (n = 84) of the isolates originated from invasive samples/invasive devices (blood, punctate, ascites, cannula, drain, broncho-alveolar lavage), 57% (n = 239) from other clinical samples (urine, wound, tracheal aspirate, etc.), and 23% (n = 98) from screening samples/stool.

### 4.2. Bacterial Identification and In Vitro Susceptibility Testing for Cefiderocol

**Figure 7:**
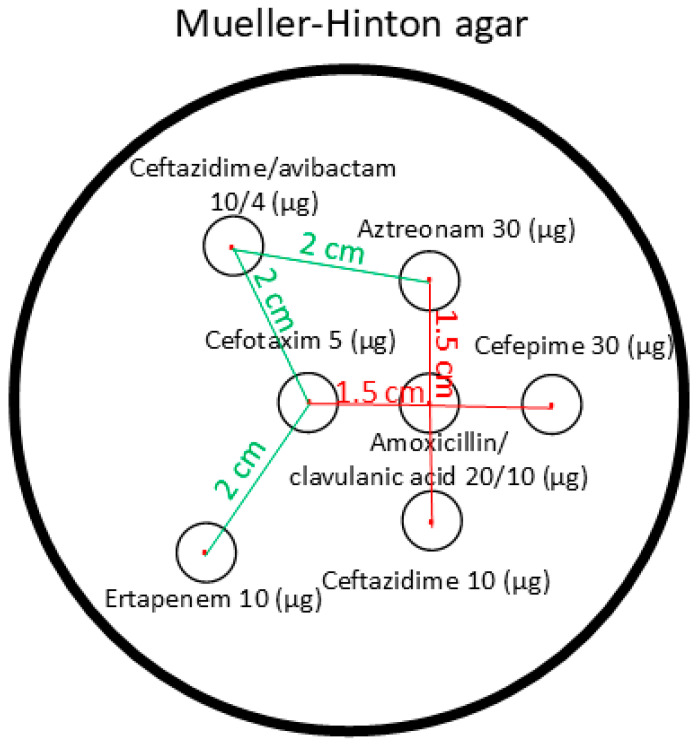
The figure shows the antibiotic-containing disks used in the synergism test and their arrangement on the Mueller–Hinton agar used for the test.

The isolates were identified using a MALDI Biotyper^®^ sirius System (Bruker, Bremen, Germany). The synergism test and the antimicrobial susceptibility test using disk diffusion were used for phenotypic screening of carbapenemase production (Mueller–Hinton agar: Bio-Rad, Marnes-la-Coquette, France; antibiotic-containing disks: MAST Diagnostica, Reinfeld, Germany) [43]. The synergism tests were performed according to a protocol developed by the National Reference Laboratory for Antimicrobial Resistance. The test was used to evaluate the synergistic effect of antibiotic discs at a specific distance from each other (Figure 7).

In vitro susceptibility testing for cefiderocol was also performed by the disk diffusion method using cefiderocol 30 µg disks (Liofilchem, Via Scozia, Italy). Tests were performed according to the EUCAST guidelines. *E. coli ATCC 25922* and *P. aeruginosa ATCC 27853* were used as quality controls [21]. The results of the susceptibility test were interpreted according to the EUCAST clinical breakpoints and instructions (v.14.0) [21,44].

### 4.3. PCR Analysis for Carbapenemase Genes

The presence of carbapenemase genes including *bla*_KPC_, *bla*_NDM_, *bla*_OXA-48-like_, and *bla*_VIM_ was detected in the multiplex PCR analysis. Multiplex PCR was performed on all isolates according to previously described protocols. The amplification was performed using a T100 Thermal Cycler (Bio-Rad) under the following thermal conditions: initial denaturation at 94 °C for 2:30 min, 33 cycles consisting of denaturation at 94 °C for 0:25 min, annealing at 59 °C for 0:40 min, elongation at 72 °C for 0:50 min, and final elongation at 72 °C for 5:00 min. The lid was at 105 °C during the run. We used the DNA primer targeting carbapenemases described by Ellington et al. (2007) [45] and Poirel et al. (2011) [46].

### 4.4. Genotyping with PFGE

Genotyping of 413 CPKP isolates was carried out using PFGE according to a previously standardized Centers for Disease Control and Prevention (CDC) protocol [47]. Strains with a Dice similarity coefficient ≥80% were considered to belong to one pulsotype. Pulsotypes showed a high correlation with MLST [48,49]. The PFGE pulsotypes are named according to the national PFGE database. After matching the WGS-based MLST and PFGE pulsotypes, we assigned a sequence type to the PFGE pulsotypes based on our database for the strains without WGS.

### 4.5. Genome-Based Typing

Ninety-eight non-duplicate isolates were selected for WGS based on PFGE results and geographical distribution. Genomic DNA was extracted from overnight cultures using the DNeasy Ultraclean Microbial Kit (Qiagen, Hilden, Germany). Genomic DNA libraries were prepared with DNA Prep Kit (Illumina, San Diego, CA, USA). WGS was performed on the Illumina MiSeq platform (150 bp paired-end). All operations were carried out according to the manufacturer’s instructions. If the contigs had a sequencing depth over 50-fold and the total size matched the expected size of around 5.5 ± 0.3 Mb with an N50 greater than 100 kbp, then we considered the draft genome assemblies suitable for downstream analysis. The comparative analysis of isolates was assessed with SeqSphere+ (Ridom, Münster, Germany). MLST and cgMLST were performed. Identification of carbapenemase genes was performed with the online tool ResFinder v4.1 [50,51,52] and SeqSphere+.

### 4.6. Statistical Analysis of the Cefiderocol Inhibition Zone Diameters and Carbapenemase and Clone Types of the CPKP Strains

Ordinary least squares regression models were fitted on cefiderocol inhibition zone diameters, using the different grouping variables as predictors in R (R v.4.2.0). The variables were the carbapenemase gene types (by PCR and WGS), the MLST and cgMLST types and the combinations for carbapenemase genes and specific clone type groups (PFGE pulsotypes, PFGE pulsotype-based STs, STs by WGS). Specific group–group comparisons (e.g., NDM vs. KPC carbapenemase type) were assessed by extracting the estimated marginal contrasts (EMCs) from the models with the R package emmeans [53]; EMC *p*-values were adjusted with Tukey’s method. The significant difference was considered *p* < 0.05. Group levels with sample sizes of n < 4 were omitted from analyses. Data and results are visualized using boxplots; group-wise estimated marginal means for the inhibition zone sizes are represented by the red dots, with a 95% confidence interval (red bars); statistical significance of between-group differences is visualized by the compact letter display method, i.e., groups that do not share any letter are significantly different from one another.

## 5. Conclusions

Population structure analysis of the CPKP strains during the study period showed that the higher prevalence of high-risk clones had a regional distribution in Hungary. The emergence and spread of the *bla*_OXA-232_+*bla*_NDM-5_-producing ST395 high-risk clone is of concern and raises the possibility of interregional transmission already described in Europe. The *K. pneumoniae* clone ST147 has been frequently found in Hungary for several years. The population structure of CPKP in Hungary has changed considerably and—as in the rest of Europe—is also determined in Hungary by the newly emerged high-risk clones.

The cefiderocol susceptibility of the different CPKP strains prevalent in Hungary can vary greatly between carbapenemase types. We observed the highest cefiderocol resistance rate and the lowest mean inhibition zone diameter for the *bla*_NDM_-carrying (especially *bla*_NDM-1_-harbouring) strains belonging to ST147 (CT7378). Our results, which are consistent with those published in the literature, suggest that the therapeutic use of cefiderocol should always be preceded by a susceptibility test and/or it is recommended to perform a molecular test to identify the carbapenemase gene. Continuous surveillance of the activity of cefiderocol is important as this agent has recently entered clinical practice.

## Figures and Tables

**Table 1:** Interpretation of cefiderocol susceptibility test results by susceptibility category.

Carbapenemase Gene	*n*	S without ATU%	S with ATU% *	R without ATU%	R with ATU% *	ATU%
*bla*_VIM_	35	86	80	13	20	14
*bla*_OXA-48-like_	57	73	56	27	44	35
*bla*_KPC_	53	63	30	27	70	28
*bla*_NDM_	153	22	30	78	70	31
*bla*_OXA-48-like_ and *bla*_NDM_	123	32	25	68	75	59

* The control isolates (ATCC 25922, ATCC 27853) complied with the EUCAST recommendation; therefore, the R or S ratio corresponds to this value, without consideration of the ATU (S ≥ 23 mm, R < 23 mm, ATU 21–23 mm) [20,21].

## Data Availability

We deposited all data described in this study in the NCBI database under BioProject PRJNA1207939. The raw data belonging to BioSample SAMN46159657 to SAMN46159754 can be found in the Sequence Read Archive (SRA) database under accessions SRX27945624 to SRX27945721.

## Supplementary Materials

The following supporting information can be downloaded at: https://www.mdpi.com/article/10.3390/antibiotics14050475/s1, Figure S1. The geographical distribution of the isolates included in the study (n = 420). Figure S2: Geographical distribution of studied CPKP strains (n = 420) in Hungary. Table S1: PFGE-based ST determination of strains without MLST (n = 315) by carbapenemase type (PCR). Table S2. *p*-values of the ordinary least squares regression model for Figure 2. Table S3. *p*-values of the ordinary least squares regression model for Figure 3. Table S4. *p*-values of the ordinary least squares regression model for Figure 4. Table S5. *p*-values of the ordinary least squares regression model for (a) part of Figure 6. Table S6. *p*-values of the ordinary least squares regression model for (b) part of Figure 6. Table S7. *p*-values of the ordinary least squares regression model for (c) part of Figure 6.
