# Supplementary material for: High Prevalence of Cefiderocol Resistance Among New Delhi Metallo-β-Lactamase Producing Klebsiella pneumoniae High-Risk Clones in Hungary
Source: Antibiotics (Basel). 2025 May 8;14(5):475. doi: 10.3390/antibiotics14050475 (PMC12108422; doi:10.3390/antibiotics14050475)
Supplement: Supplementary file 1 [file antibiotics-14-00475-s001.zip › antibiotics-3566003-supplementary.pdf]

Supplementary Materials:

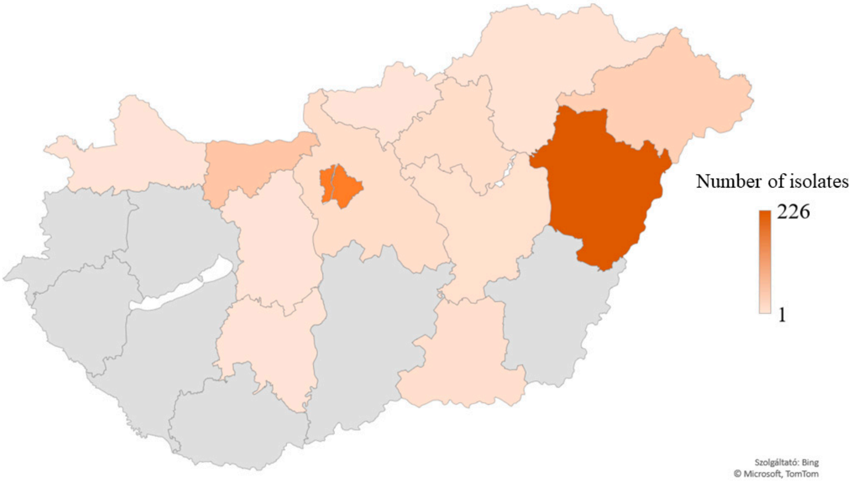

Figure S1. The geographical distribution of the isolates included in the study (n=420). The map shows the place of origin of the studied isolates, broken down by county. The scale shown in the figure indicates the min-max range (1-226) of the isolates submitted; the darkening of the colour corresponds directly to the number of submitted isolates.

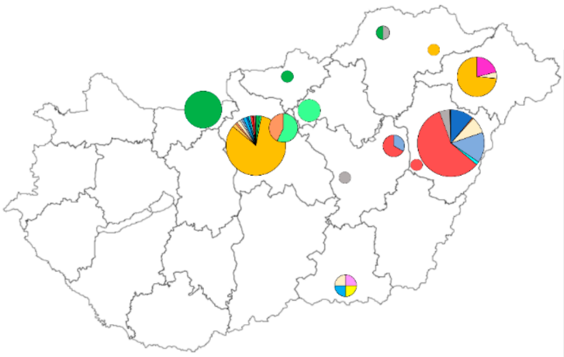

	PFGE based ST and MSLT	Carbapenemase type (by PCR)	No. of isolates
	ST395	$bla_{\text{OXA-48-like}}$+bla_{NDM}	119
	ST395	$bla_{\text{OXA-48-like}}$	31
	ST395	bla_{NDM}	20
	ST147	bla_{NDM}	147
	ST147	$bla_{\text{OXA-48-like}}$	3
	ST147	$bla_{\text{OXA-48-like}}$+bla_{NDM}	1
	ST258	bla_{KPC}	39
	ST15	bla_{VIM}	15
	ST307	bla_{KPC}	10
	ST307	$bla_{\text{OXA-48-like}}$	1
	ST11	bla_{NDM}	4
	ST15	$bla_{\text{OXA-48-like}}$	4
	ST107	bla_{NDM}	4
	ST15	bla_{NDM}	3
	ST1198	$bla_{\text{OXA-48-like}}$	1
	ST383	$bla_{\text{OXA-48-like}}$+bla_{NDM}	1
	ST219	bla_{NDM}	1
	ST17	bla_{VIM}	1

Figure S2. Geographical distribution of studied CPKP strains (n=420) in Hungary. In the figure, the different colours represent the different STs of the individual categorized strains indicated in the column "PFGE based ST and MLST". The size of the pie charts corresponds to the number of isolates. The column "Carbapenemase type" shows the types of the carbapenemase genes determined by PCR.

Table S1. PFGE-based ST determination of strains without MLST (n=315) by carbapenemase type (PCR).

PFGE pulsotype	Carbapenemase gene by PCR	PFGE based ST							Missing	No. of HCIs
		ST 11	ST 15	ST 107	ST 147	ST 258	ST 307	ST 395		
G	bla_{VIM}								1	
KP027	$bla_{\text{OXA-48-like}}$								2	
	$bla_{\text{OXA-48-like}}$+ bla_{NDM}								1	
KP053	bla_{NDM}	2								2
KP197	bla_{KPC}						8			3
KP205	bla_{VIM}								1	
KP234	bla_{VIM}								1	
KP294	bla_{VIM}								1	
KP297	$bla_{\text{OXA-48-like}}$								9	
	bla_{VIM}								1	
KP372	bla_{NDM}				15					4
KP387	$bla_{\text{OXA-48-like}}$								1	
KP388	bla_{VIM}								1	
KP393	bla_{VIM}								2	
KP408	bla_{VIM}								1	
KP410	bla_{VIM}								3	
KP413	bla_{NDM}							16		3
	$bla_{\text{OXA-48-like}}$							20		2
	$bla_{\text{OXA-48-like}}$+ bla_{NDM}							99		3
KP417	bla_{VIM}								1	
KP427	bla_{NDM}								1	
KP428	$bla_{\text{OXA-48-like}}$								1	
KP429	bla_{KPC}								1	
KP430	bla_{VIM}								1	
KP431	bla_{NDM}			3						1
KP436	bla_{NDM}								1	
KP438	bla_{NDM}								1	
KP439	bla_{NDM}								2	
KP443	bla_{VIM}								1	
KP444	bla_{VIM}								1	
KP445	$bla_{\text{OXA-48-like}}$								1	
KP446	$bla_{\text{OXA-48-like}}$								1	
KP447	bla_{VIM}								1	
KP452	bla_{KPC}								1	
N	bla_{NDM}		3							3
	$bla_{\text{OXA-48-like}}$		4							2
	bla_{VIM}		15							5
R	bla_{NDM}				69					12
	$bla_{\text{OXA-48-like}}$				2					2
	$bla_{\text{OXA-48-like}}$+ bla_{NDM}				1					1
S	bla_{KPC}					18				4

Table S2. P-values of the ordinary least squares regression model for Figure 2.

contrast	estimate	SE	df	t.ratio	p.value
KPC - NDM	0.8979571	0.4562081	366	1.9683061	0.2836604
KPC - (OXA-48-like)	-2.6168402	0.5893301	366	-4.4403642	0.0001158
KPC - (OXA-48-like+NDM)	-0.0835047	0.4688634	366	-0.1781003	0.9997742
KPC - VIM	-2.7168400	0.7963249	366	-3.4117230	0.0064121
NDM - (OXA-48-like)	-3.5147973	0.4932008	366	-7.1265035	0.0000000
NDM - (OXA-48-like+NDM)	-0.9814618	0.3402499	366	-2.8845325	0.0335835
NDM - VIM	-3.6147971	0.7280594	366	-4.9649758	0.0000104
(OXA-48-like) - (OXA-48-like+NDM)	2.5333355	0.5049298	366	5.0172032	0.0000081
(OXA-48-like) - VIM	-0.0999998	0.8180798	366	-0.1222372	0.9999495
(OXA-48-like+NDM) - VIM	-2.6333353	0.7360554	366	-3.5776320	0.0035903

Table S3. P-values of the ordinary least squares regression model for Figure 3.

contrast	estimate	SE	df	t.ratio	p.value
KPC & KP197 - KPC & S type	-0.2000003	0.9538015	345	-0.2096876	1.0000000
KPC & KP197 - NDM & KP053	-2.4500036	1.5876808	345	-1.5431336	0.9273664
KPC & KP197 - NDM & KP372	1.8263155	1.0484588	345	1.7419049	0.8473955
KPC & KP197 - NDM & KP413	-0.4894733	1.0484588	345	-0.4668503	0.9999988
KPC & KP197 - NDM & KP431	-2.2000123	1.5876808	345	-1.3856767	0.9656645
KPC & KP197 - NDM & R type	0.9979171	0.8917569	345	1.1190461	0.9936928
KPC & KP197 - (OXA-48-like & KP297)	-0.5000017	1.4699068	345	-0.3401588	1.0000000
KPC & KP197 - (OXA-48-like & KP413)	-2.7769238	0.9986050	345	-2.7808029	0.1929415
KPC & KP197 - (OXA-48-like & N type)	-2.9499862	1.5876808	345	-1.8580474	0.7841080
KPC & KP197 - (OXA-48-like+NDM & KP413)	-0.2169497	0.8838797	345	-0.2454516	1.0000000
KPC & KP197 - VIM & N type	-2.6999993	1.0956038	345	-2.4643938	0.3661604
KPC & S type - NDM & KP053	-2.2500033	1.4106914	345	-1.5949649	0.9100676
KPC & S type - NDM & KP372	2.0263158	0.7540463	345	2.6872566	0.2370988
KPC & S type - NDM & KP413	-0.2894730	0.7540463	345	-0.3838929	0.9999998
KPC & S type - NDM & KP431	-2.0000120	1.4106914	345	-1.4177530	0.9594723
KPC & S type - NDM & R type	1.1979174	0.5143446	345	2.3290173	0.4580269
KPC & S type - (OXA-48-like & KP297)	-0.3000014	1.2766934	345	-0.2349831	1.0000000
KPC & S type - (OXA-48-like & KP413)	-2.5769235	0.6830314	345	-3.7727744	0.0101849
KPC & S type - (OXA-48-like & N type)	-2.7499859	1.4106914	345	-1.9493887	0.7270498

KPC & S type - (OXA-48-like+NDM & KP413)	-0.0169494	0.5005629	345	-0.0338607	1.0000000
KPC & S type - VIM & N type	-2.4999990	0.8183323	345	-3.0549926	0.0977197
NDM & KP053 - NDM & KP372	4.2763191	1.4763397	345	2.8965685	0.1467502
NDM & KP053 - NDM & KP413	1.9605302	1.4763397	345	1.3279669	0.9749904
NDM & KP053 - NDM & KP431	0.2499913	1.8976415	345	0.1317379	1.0000000
NDM & KP053 - NDM & R type	3.4479207	1.3695048	345	2.5176405	0.3324929
NDM & KP053 - (OXA-48-like & KP297)	1.9500019	1.8002608	345	1.0831774	0.9952263
NDM & KP053 - (OXA-48-like & KP413)	-0.3269202	1.4413622	345	-0.2268134	1.0000000
NDM & KP053 - (OXA-48-like & N type)	-0.4999826	1.8976415	345	-0.2634758	1.0000000
NDM & KP053 - (OXA-48-like+NDM & KP413)	2.2330539	1.3643886	345	1.6366700	0.8943366
NDM & KP053 - VIM & N type	-0.2499957	1.5101857	345	-0.1655397	1.0000000
NDM & KP372 - NDM & KP413	-2.3157888	0.8706976	345	-2.6596935	0.2512821
NDM & KP372 - NDM & KP431	-4.0263278	1.4763397	345	-2.7272367	0.2174720
NDM & KP372 - NDM & R type	-0.8283984	0.6738538	345	-1.2293444	0.9863009
NDM & KP372 - (OXA-48-like & KP297)	-2.3263172	1.3488790	345	-1.7246300	0.8558055
NDM & KP372 - (OXA-48-like & KP413)	-4.6032393	0.8099756	345	-5.6831824	0.0000018
NDM & KP372 - (OXA-48-like & N type)	-4.7763017	1.4763397	345	-3.2352322	0.0589372
NDM & KP372 - (OXA-48-like+NDM & KP413)	-2.0432652	0.6633942	345	-3.0800168	0.0913396
NDM & KP372 - VIM & N type	-4.5263148	0.9269284	345	-4.8831330	0.0001009
NDM & KP413 - NDM & KP431	-1.7105389	1.4763397	345	-1.1586351	0.9915548
NDM & KP413 - NDM & R type	1.4873904	0.6738538	345	2.2072896	0.5454892
NDM & KP413 - (OXA-48-like & KP297)	-0.0105284	1.3488790	345	-0.0078053	1.0000000
NDM & KP413 - (OXA-48-like & KP413)	-2.2874504	0.8099756	345	-2.8240979	0.1745866
NDM & KP413 - (OXA-48-like & N type)	-2.4605128	1.4763397	345	-1.6666306	0.8820262
NDM & KP413 - (OXA-48-like+NDM & KP413)	0.2725236	0.6633942	345	0.4108020	0.9999997
NDM & KP413 - VIM & N type	-2.2105260	0.9269284	345	-2.3847860	0.4192431
NDM & KP431 - NDM & R type	3.1979294	1.3695048	345	2.3350991	0.4537449
NDM & KP431 - (OXA-48-like & KP297)	1.7000106	1.8002608	345	0.9443135	0.9985920
NDM & KP431 - (OXA-48-like & KP413)	-0.5769115	1.4413622	345	-0.4002544	0.9999998
NDM & KP431 - (OXA-48-like & N type)	-0.7499739	1.8976415	345	-0.3952137	0.9999998

NDM & KP431 - (OXA-48-like+NDM & KP413)	1.9830626	1.3643886	345	1.4534441	0.9516604
NDM & KP431 - VIM & N type	-0.4999870	1.5101857	345	-0.3310765	1.0000000
NDM & R type - (OXA-48-like & KP297)	-1.4979188	1.2310317	345	-1.2167995	0.9873854
NDM & R type - (OXA-48-like & KP413)	-3.7748409	0.5933170	345	-6.3622665	0.0000000
NDM & R type - (OXA-48-like & N type)	-3.9479033	1.3695048	345	-2.8827232	0.1517938
NDM & R type - (OXA-48-like+NDM & KP413)	-1.2148668	0.3688582	345	-3.2935877	0.0495694
NDM & R type - VIM & N type	-3.6979164	0.7450912	345	-4.9630388	0.0000693
(OXA-48-like & KP297) - (OXA-48-like & KP413)	-2.2769221	1.3105040	345	-1.7374401	0.8495951
(OXA-48-like & KP297) - (OXA-48-like & N type)	-2.4499845	1.8002608	345	-1.3609053	0.9699413
(OXA-48-like & KP297) - (OXA-48-like+NDM & KP413)	0.2830520	1.2253375	345	0.2309992	1.0000000
(OXA-48-like & KP297) - VIM & N type	-2.1999976	1.3858414	345	-1.5874815	0.9127182
(OXA-48-like & KP413) - (OXA-48-like & N type)	-0.1730624	1.4413622	345	-0.1200686	1.0000000
(OXA-48-like & KP413) - (OXA-48-like+NDM & KP413)	2.5599741	0.5814103	345	4.4030420	0.0008600
(OXA-48-like & KP413) - VIM & N type	0.0769245	0.8701393	345	0.0884048	1.0000000
(OXA-48-like & N type) - (OXA-48-like+NDM & KP413)	2.7330364	1.3643886	345	2.0031217	0.6911018
(OXA-48-like & N type) - VIM & N type	0.2499869	1.5101857	345	0.1655339	1.0000000
(OXA-48-like+NDM & KP413) - VIM & N type	-2.4830496	0.7356451	345	-3.3753362	0.0386065

Table S4. P-values of the ordinary least squares regression model for Figure 4.

contrast	estimate	SE	df	t.ratio	p.value
KPC & 258 - KPC & 307	0.1358975	0.9596705	349	0.1416085	1.0000000
KPC & 258 - NDM & 107	-2.0641024	1.4214394	349	-1.4521213	0.9095422
KPC & 258 - NDM & 11	-2.3141023	1.4214394	349	-1.6279993	0.8336827
KPC & 258 - NDM & 147	1.2706799	0.5016901	349	2.5327984	0.2547795
KPC & 258 - NDM & 395	-0.1141026	0.7446207	349	-0.1532358	1.0000000
KPC & 258 - (OXA-48-like & 15)	-2.8141023	1.4214394	349	-1.9797553	0.6138751
KPC & 258 - (OXA-48-like & 395)	-2.2737800	0.6514668	349	-3.4902468	0.0191866

KPC & 258 - (OXA-48-like+NDM & 395)	-0.0810516	0.5000725	349	-0.1620796	1.0000000
KPC & 258 - VIM & 15	-2.5641023	0.8225747	349	-3.1171664	0.0607497
KPC & 307 - NDM & 107	-2.1999999	1.6017342	349	-1.3735112	0.9345574
KPC & 307 - NDM & 11	-2.4499998	1.6017342	349	-1.5295920	0.8795174
KPC & 307 - NDM & 147	1.1347825	0.8926116	349	1.2713060	0.9593554
KPC & 307 - NDM & 395	-0.2500001	1.0485812	349	-0.2384175	1.0000000
KPC & 307 - (OXA-48-like & 15)	-2.9499997	1.6017342	349	-1.8417536	0.7080393
KPC & 307 - (OXA-48-like & 395)	-2.4096774	0.9846174	349	-2.4473237	0.3013012
KPC & 307 - (OXA-48-like+NDM & 395)	-0.2169490	0.8917034	349	-0.2432973	0.9999999
KPC & 307 - VIM & 15	-2.6999998	1.1053016	349	-2.4427720	0.3039074
NDM & 107 - NDM & 11	-0.2500000	1.9144385	349	-0.1305866	1.0000000
NDM & 107 - NDM & 147	3.3347823	1.3770540	349	2.4216786	0.3161490
NDM & 107 - NDM & 395	1.9499998	1.4829177	349	1.3149751	0.9497744
NDM & 107 - (OXA-48-like & 15)	-0.7499999	1.9144385	349	-0.3917597	0.9999964
NDM & 107 - (OXA-48-like & 395)	-0.2096776	1.4383998	349	-0.1457714	1.0000000
NDM & 107 - (OXA-48-like+NDM & 395)	1.9830508	1.3764655	349	1.4406832	0.9135194
NDM & 107 - VIM & 15	-0.5000000	1.5235531	349	-0.3281802	0.9999992
NDM & 11 - NDM & 147	3.5847823	1.3770540	349	2.6032255	0.2200362
NDM & 11 - NDM & 395	2.1999998	1.4829177	349	1.4835616	0.8980071
NDM & 11 - (OXA-48-like & 15)	-0.4999999	1.9144385	349	-0.2611731	0.9999999
NDM & 11 - (OXA-48-like & 395)	0.0403224	1.4383998	349	0.0280328	1.0000000
NDM & 11 - (OXA-48-like+NDM & 395)	2.2330508	1.3764655	349	1.6223078	0.8365616
NDM & 11 - VIM & 15	-0.2500000	1.5235531	349	-0.1640901	1.0000000
NDM & 147 - NDM & 395	-1.3847825	0.6559329	349	-2.1111650	0.5208521

NDM & 147 - (OXA-48-like & 15)	-4.0847822	1.3770540	349	-2.9663194	0.0920674
NDM & 147 - (OXA-48-like & 395)	-3.5444599	0.5479022	349	-6.4691465	0.0000000
NDM & 147 - (OXA-48-like+NDM & 395)	-1.3517315	0.3547680	349	-3.8101842	0.0062699
NDM & 147 - VIM & 15	-3.8347823	0.7432477	349	-5.1594946	0.0000183
NDM & 395 - (OXA-48-like & 15)	-2.6999997	1.4829177	349	-1.8207347	0.7216961
NDM & 395 - (OXA-48-like & 395)	-2.1596774	0.7765075	349	-2.7812704	0.1469615
NDM & 395 - (OXA-48-like+NDM & 395)	0.0330510	0.6546964	349	0.0504830	1.0000000
NDM & 395 - VIM & 15	-2.4499998	0.9247617	349	-2.6493310	0.1990911
(OXA-48-like & 15) - (OXA-48-like & 395)	0.5403223	1.4383998	349	0.3756413	0.9999975
(OXA-48-like & 15) - (OXA-48-like+NDM & 395)	2.7330507	1.3764655	349	1.9855570	0.6097970
(OXA-48-like & 15) - VIM & 15	0.2499999	1.5235531	349	0.1640901	1.0000000
(OXA-48-like & 395) - (OXA-48-like+NDM & 395)	2.1927284	0.5464214	349	4.0128889	0.0029148
(OXA-48-like & 395) - VIM & 15	-0.2903224	0.8515475	349	-0.3409351	0.9999989
(OXA-48-like+NDM & 395) - VIM & 15	-2.4830508	0.7421567	349	-3.3457231	0.0305975

Table S5. P-values of the ordinary least squares regression model for a) part of Figure 6.

contrast	estimate	SE	df	t.ratio	p.value
KPC & 258 - NDM & 147	1.7388657	0.6144794	81	2.8298195	0.0451624
KPC & 258 - NDM & 395	1.3904759	1.0819026	81	1.2852136	0.7009804
KPC & 258 - OXA & 395	-1.9004279	0.8092172	81	-2.3484768	0.1404961
KPC & 258 - (OXA+NDM & 395)	-0.2305760	0.6883981	81	-0.3349457	0.9972335
NDM & 147 - NDM & 395	-0.3483898	1.0478076	81	-0.3324940	0.9973114
NDM & 147 - OXA & 395	-3.6392935	0.7630335	81	-4.7695072	0.0000774

NDM & 147 - (OXA+NDM & 395)	-1.9694416	0.6334662	81	-3.1089922	0.0212201
NDM & 395 - OXA & 395	-3.2909038	1.1726672	81	-2.8063408	0.0479761
NDM & 395 - (OXA+NDM & 395)	-1.6210519	1.0927981	81	-1.4833955	0.5761389
OXA & 395 - (OXA+NDM & 395)	1.6698519	0.8237276	81	2.0271895	0.2625395

Table S6. P-values of the ordinary least squares regression model for b) part of Figure 6.

contrast	estimate	SE	df	t.ratio	p.value
CT_cgMLST1163 - CT_cgMLST5854	0.6715934	0.6394472	74	1.0502719	0.7205192
CT_cgMLST1163 - CT_cgMLST6235	0.0620689	1.0806468	74	0.0574368	0.9999313
CT_cgMLST1163 - CT_cgMLST7378	2.6537361	0.6158311	74	4.3091946	0.0002857
CT_cgMLST5854 - CT_cgMLST6235	-0.6095245	1.1105045	74	-0.5488717	0.9465727
CT_cgMLST5854 - CT_cgMLST7378	1.9821427	0.6668364	74	2.9724573	0.0203510
CT_cgMLST6235 - CT_cgMLST7378	2.5916672	1.0970759	74	2.3623408	0.0935263

Table S7. P-values of the ordinary least squares regression model for c) part of Figure 6.

contrast	estimate	SE	df	t.ratio	p.value
(KPC-2) - (NDM-1)	1.4960506	0.6334201	87	2.3618616	0.1813876
(KPC-2) - (NDM-5)	0.6904909	1.2584781	87	0.5486713	0.9938990
(KPC-2) - (OXA-232)	-2.9761798	1.0678541	87	-2.7870659	0.0691536
(KPC-2) - (OXA-232+NDM-5)	-0.2305767	0.7303968	87	-0.3156869	0.9995679
(KPC-2) - (OXA-48)	-1.9345245	0.9584284	87	-2.0184341	0.3405196
(NDM-1) - (NDM-5)	-0.8055597	1.2158054	87	-0.6625729	0.9855144
(NDM-1) - (OXA-232)	-4.4722304	1.0172158	87	-4.3965406	0.0004340
(NDM-1) - (OXA-232+NDM-5)	-1.7266273	0.6541368	87	-2.6395507	0.0987421
(NDM-1) - (OXA-48)	-3.4305751	0.9016654	87	-3.8047098	0.0034845

(NDM-5) - (OXA-232)	-3.6666706	1.4890514	87	-2.4624205	0.1469609
(NDM-5) - (OXA-232+NDM-5)	-0.9210676	1.2690316	87	-0.7258035	0.9782530
(NDM-5) - (OXA-48)	-2.6250154	1.4126382	87	-1.8582362	0.4346764
(OXA-232) - (OXA-232+NDM-5)	2.7456031	1.0802714	87	2.5415863	0.1235447
(OXA-232) - (OXA-48)	1.0416552	1.2458298	87	0.8361136	0.9599148
(OXA-232+NDM-5) - (OXA-48)	-1.7039478	0.9722443	87	-1.7525923	0.5014713
